# Whole genome sequencing uncovers a novel IND-16 metallo-β-lactamase from an extensively drug-resistant *Chryseobacterium indologenes* strain J31

**DOI:** 10.1186/s13099-016-0130-4

**Published:** 2016-10-21

**Authors:** Tingting Wang, Xiawei Jiang, Chunyan Feng, Ang Li, Huihui Dong, Shaoqiang Wu, Beiwen Zheng

**Affiliations:** 1 State Key Laboratory for Diagnosis and Treatment of Infectious Diseases, Collaborative Innovation Center for Diagnosis and Treatment of Infectious Diseases, The First Affiliated Hospital, School of Medicine, Zhejiang University, Hangzhou, 310003 China; 2 MOE Key Laboratory of Bioinformatics and Bioinformatics Division, TNLIST/Department of Automation, Center for Synthetic and Systems Biology, Tsinghua University, Beijing, 100084 China; 3 College of Basic Medical Sciences, Zhejiang Chinese Medical University, Hangzhou, 310053 China; 4 Institute of Animal Quarantine, Chinese Academy of Inspection and Quarantine, Beijing, 100029 China

**Keywords:** *Chryseobacterium indologenes*, Extensively antibiotic resistant, Whole genome sequencing, IND-16, Comparative genomic analysis

## Abstract

**Background:**

*Chryseobacterium indologenes* is an emerging opportunistic pathogen in hospital-acquired infection, which is intrinsically resistant to most antimicrobial agents against gram-negative bacteria. In the purpose of extending our understanding of the resistance mechanism of *C. indologenes*, we sequenced and analyzed the genome of an extensively antibiotic resistant *C. indologenes* strain, isolated from a Chinese prostate cancer patient. We also investigated the presence of antibiotic resistance genes, particularly metallo-β-lactamase (MBL) genes, and performed a comparative genomic analysis with other *Chryseobacterium* species.

**Results:**

16s rRNA sequencing indicated the isolate belongs to *C. indologenes*. We assembled a total of 1095M bp clean-filtered reads into 171 contigs by de novo assembly. The draft genome of *C. indologenes* J31 consisted of 5,830,795 bp with a GC content of 36.9 %. RAST analysis revealed the genome contained 5196 coding sequences (CDSs), 28 rRNAs, 81 tRNAs and 114 pseudogenes. We detected 90 antibiotic resistance genes from different drug classes in the whole genome. Notably, a novel *bla*_IND_ allele *bla*_IND-16_ was identified, which shared 99 % identity with *bla*_IND-8_ and *bla*_IND-10_. By comparing strain J31 genome to the closely four related neighbors in the genus *Chryseobacterium*, we identified 2634 conserved genes, and 1449 unique genes.

**Conclusions:**

In this study, we described the whole genome sequence of *C. indologenes* strain J31. Numerous resistance determinants were detected in the genome and might be responsible for the extensively antibiotic resistance of this strain. Comparative genomic analysis revealed the presence of considerable strain-specific genes which would contribute to the distinctive characteristics of strain J31. Our study provides the insight of the multidrug resistance mechanism in genus *Chryseobacterium*.

**Electronic supplementary material:**

The online version of this article (doi:10.1186/s13099-016-0130-4) contains supplementary material, which is available to authorized users.

## Background

*Chryseobacterium indologenes*, of which the taxonomic characteristics were first described in 1983 [1], is a common gram-negative bacterium and belongs to the genus *Chryseobacterium*. It is widely found existing in natural and nosocomial environment and is occasionally isolated from the human gut [2]. As an uncommon pathogen, it usually causes opportunistic infection to the susceptible populations such as the infant, the elder, the immuno-suppressed patient and the long-term inpatient [3]. Since the first case of location outbreak emerged in Taiwan, China [4], *C. indologenes* has been documented to cause invasive infections, which usually result in bacteremia or pneumonia with high mortality [5–7].

*Chryseobacterium indologenes* has been proved to be intrinsically resistant to most antimicrobial agents often used to treat gram-negative bacteria [8, 9], but the mechanism of the multidrug resistance is not clear. The metallo-β-lactamase (MBL), which can cleave the β-lactam ring of antibiotics of the penicillin family, is thought to be closely associated with multidrug resistance of this bacterium [10]. Since the first type of class B MBL gene, *bla*_IND_, was identified in *C. indologenes*, there are fifteen IND variants have been deposited in Genbank to date. These enzymes share 27–92 % identity with that of IND-1 at the amino acid sequence level [11–14]. The investigation of new *bla*_IND_ allele would extend our understanding of the resistance mechanism of this bacterium.

The genomic information would provide more details about antibiotic resistance genes and help decipher the antibiotic resistance. However, only three whole genome sequences of different *C. indologenes* strains have been deposited in the NCBI genome database (https://www.ncbi.nlm.nih.gov/genome). In this study, we reported the whole genome sequence of one *C. indologenes* strain isolated from a prostate cancer patient in China and analyzed the multidrug resistance at the genomic level.

## Methods

### Strain information, antimicrobial susceptibility testing, DNA isolation and 16S rRNA sequencing

The urine sample was from a patient with prostate cancer in June 2014. The isolate was cultured aerobically on Columbia blood agar base plate at 37 °C. Susceptibility testing for ampicillin, amikacin, ciprofloxacin, levofloxacin, cefoperazone, nitrofurantoin, imipenem, iobramycin, piperacillin-tazobactam, colistin, cephalosporins and trimethoprim-sulfamethoxazole (TMP-SMZ) were determined by the disc diffusion method and interpreted concerning the CLSI guidelines. Late log phase cells were harvested, and genomic DNA was extracted using a DNeasy Blood & Tissue Kit (Qiagen, Germany) according to the manufacturer’s instruction. In the purpose of taxonomic identification, we amplified the 16S rRNA gene with a 16S rRNA universal primer set and the PCR product was sequenced as previously [15]. A neighbor-joining phylogenetic tree was constructed based on the Tamura-Nei model using MEGA6 (http://www.megasoftware.net/).

### Genome sequencing, assembly, and annotation

We conducted genome sequencing, assembly, and annotation by following previous studies [16, 17]. In brief, whole genome sequencing was performed using an Illumina HiSeq 2000 genomic sequencer with a 2 × 100 paired-end sequencing strategy (DNA libraries was in a size of 500-bp insertion). We removed all reads with adaptor contamination and with unknown nucleotides comprising more than 5 %. Then, low-quality reads with ambiguous sequence “N” were discarded. Subsequently, clean-filtered reads were de novo assembled into scaffolds using Velvet 1.2.07 [18]. We used VelvetOptimiser for automatically optimizing optimum k-Mer value. And then PAGIT (Post-Assembly Genome Improvement Toolkit) [19] was used to extend the initial contigs and correct sequencing errors. Open reading frames (ORFs), tRNAs and rRNAs were identified using Glimmer version 3.0 [20], tRNAscan-SE [21] and RNAmmer [22], respectively. The functional genes were annotated and classified using the RAST (rapid annotation using subsystem technology) server [23] and the COGs (clusters of orthologous groups of proteins) databases [24]. We predicted the plasmid replicons by the PlasmidFinder Tool (https://cge.cbs.dtu.dk/services/PlasmidFinder/). ISfinder (https://www-is.biotoul.fr/blast.php) was employed to search the IS sequences in the genome, with an e value of 1E−3.

### Antibiotic resistance genes prediction and virulence factors analysis of J31

The protein-coding sequences were annotated by antibiotic resistance database (ARDB) [25] and antibiotic resistance gene-ANNOTation (ARG-ANNOT) with default parameters [26]. We further verified all these putative ARGs through a BLAST search with cut-off e value of 1E−0.5. Using PCR sequencing, we verified IND-16 gene and downloaded other IND protein sequences from NCBI website. Multiple sequence alignments of the amino acid sequences were performed using Clustal Omega (http://www.ebi.ac.uk/Tools/msa/clustalo), and then the alignment results were visualized using BoxShade (http://www.ch.embnet.org/software/BOX_form.html). The virulence factors were predicted by VFDB database (http://www.mgc.ac.cn/VFs/main.htm) using BLAST with an e value threshold of 1E−5.

### Comparative analysis

Comparative genomic analysis was performed by orthology identification method as we described previously [15, 17]. We downloaded the genome sequences used in the comparative analysis, from NCBI genome database. BLASTN were used for aligning the whole genomes between strain J31 and other five *Chryseobacterium* species and then genome alignment visualization was performed using BLAST ring image generator (BRIG) [27].

### Quality assurance

We isolated a single colony of strain J31 and purified the genomic DNA from a pure culture of the isolate. The strain persevered in the State Key Laboratory for Diagnosis and Treatment of Infectious Diseases, Zhejiang University. The genome confirmed to *C. indologenes* by 16S rRNA sequencing. We constructed a phylogenetic tree from the 16s rRNA sequences and the position classified to *C. indologenes.* We assessed the potential contamination of the genomic library by other microorganisms by using a BLAST search against the non-redundant database.

## Results

### Identification of strain J31

**Fig. 1:**
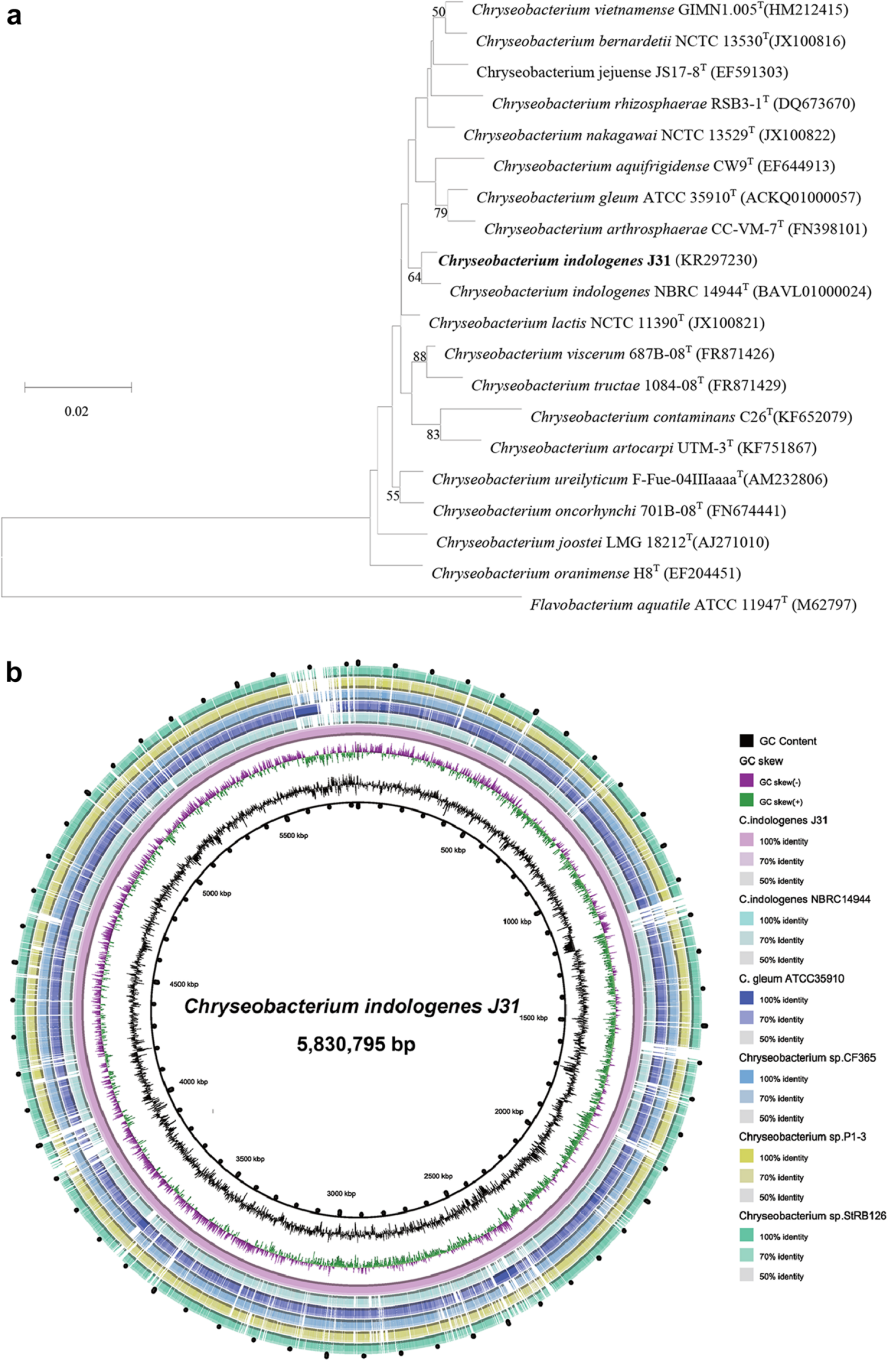
**a** 16S rRNA-based phylogenetic analysis of *C*. *indologene* J31. Phylogenetic tree highlighted the position of *C*. *indologene* J31 within the genus *Chryseobacterium*. The strains and their corresponding GenBank accession numbers for 16S rRNA genes were shown following the organism names. *Flavobacterium aquatile* ATCC 11947^T^ was used as out group. **b** Graphical circular genomic map of Peptoclostridium *C*. *indologene* J31 using CGView. The *inner circles* were GC content and GC skew of the *C*. *indologene* J31. The *outer rings* showed the BLASTN atlas of the genomes of *Chryseobacterium* isolates. The strains were *C*. *indologene* NBRC 14944 (BAVL01000000), *C. gleum* ATCC 35910 (ACKQ02000000), *Chryseobacterium* sp. CF365 (JQJM01000000), *Chryseobacterium* sp. P1-3 (JPEQ01000000) and *Chryseobacterium* sp. StRB126 (NZ_AP014624.1). The *white* and *colored regions* of the *outer rings* indicated regions absent and present, respectively

We aligned the 16S rRNA gene sequence of strain J31 to the nucleotide sequences within the NCBI-NR/NT database by using BLASTN, to identify the taxonomic status of the strain. The sequence revealed 99 % sequence similarity to the members of genus *Chryseobacterium*. Phylogenetic tree indicated the strain J31 was clearly classified into the same branch of the strain *C. indologenes* NBRC 14944 (Fig. 1a).

### General genome properties

We performed whole genome sequencing by Illumina HiSeq 2000 system with 2 × 100 bp paired-end in length. After quality control, we assembled the filtered 1095M bp reads into contigs. The draft genome sequence of *C. indologenes* strain J31 revealed a total size of 5,830,795 bp and a GC content of 36.9 %. The assembled genome covered an average depth of 187.8-fold and contained 171 contigs, of which the largest one consisted of 476,013 bp and the length of N50 was 106,549 bp. Annotation of the contigs identified 5196 coding sequences (CDSs), 28 rRNAs, 81 tRNAs and 114 pseudogenes. BLASTN search between strain J31 and other five *Chryseobacterium* species revealed high similarity between *Chryseobacterium* isolates (Fig. 1b).

**Fig. 2:**
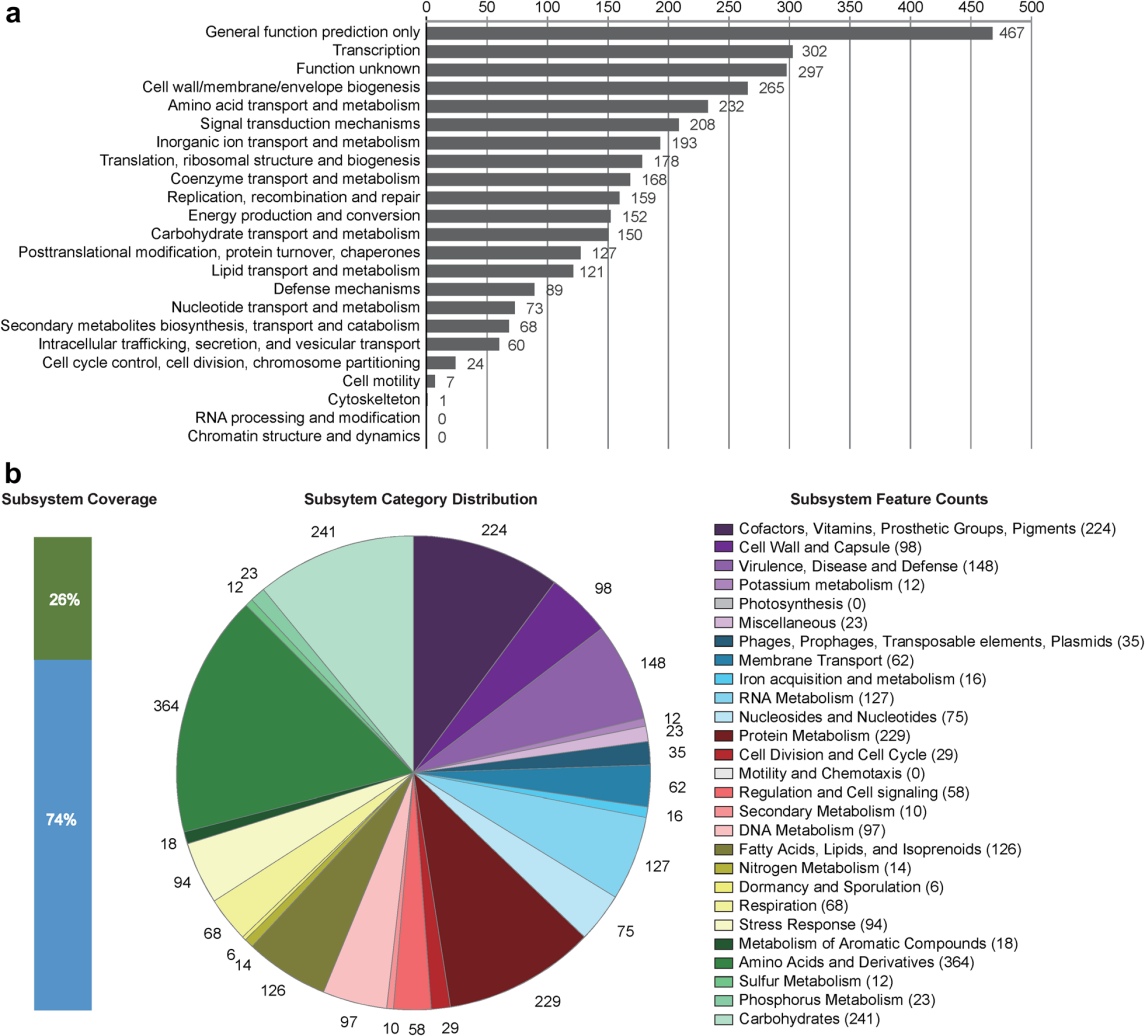
Statistics of COGs analysis and subsystem annotation of *C*. *indologene* J31. **a** COGs distribution. Each *bar* indicated the number of annotated genes based on COG database; **b** subsystem category distribution. The *green bar* represented the percentage of proteins that could be annotated by RAST server, while the *blue bar* indicated the proteins not be annotated. The *pie chart* demonstrated the abundance of each subsystem category and the count of each subsystem feature was shown

Next, we predicted gene functions using COG annotations and RAST analysis. We categorized a total of 2921 genes into COG functional groups, including putative or hypothetical genes and gene of unknown functions. According to the COG distribution, genes associated with transcription (302 ORFs), cell wall/membrane/envelope biogenesis (265 ORFs), amino acid transport and metabolism (232 ORFs), and signal transduction mechanisms (208 ORFs) are the abundant categories (Fig. 2a). For the RAST annotations, we could only annotate 1417 ORFs in the subsystems which consisted of 1369 non-hypothetical genes and 48 hypothetical genes, while we did not find the other 4034 ORFs in the subsystems (Fig. 2b). The low subsystem coverage might indicate the gene functions of *C. indologenes* strain J31 remains to be explored. For the subsystem distribution, genes responsible for amino acids and derivatives (364 ORFs), carbohydrates (241 ORFs), cofactors, vitamins, prosthetic groups, pigments (224 ORFs) and protein metabolism (228 ORFs) are the abundant genes categories (Fig. 2b).

### Antimicrobial susceptibility profiles and antibiotic resistance genes

The in vitro antimicrobial susceptibility testing demonstrates that the strain J31 is only susceptible to TMP-SMZ, but resistant to all the other tested antibiotics including ampicillin, amikacin, ciprofloxacin, levofloxacin, cefoperazone, nitrofurantoin, imipenem, iobramycin, piperacillin-tazobactam, colistin and cephalosporins (Additional file 1: Table S1). We screened the antibiotic resistance genes (ARGs) in the genome-wide scale in order to further explore the genetic basis of extensive resistance in this strain. In silico analysis revealed the presence of a considerable number of putative ARGs from different drug classes, 90 genes in total (Additional file 2: Table S2), which was more than that observed in *Chryseobacterium oranimense* [16]. This isolate encoded 30 β-lactamase genes, which included amber class A, MBL, class C, and other novel β-lactamases genes. These ARGs might confer high-level resistance to cefepime, cefoperazone and piperacillin-tazobactam, which have been active in previous studies [11, 28]. In addition, we detected genes corresponding to rifampin, aminoglycosides, phenicols, sulfonamide, macrolide and trimethoprim resistance, which are consistent with the phenotypic results (Additional file 2: Table S2). Moreover, Multidrug efflux pumps including ABC-type transporter, MFS superfamily transporter and RND family transporter are also scattered among the genome. Therefore, these predicted ARGs might contribute to the multidrug resistance of stain J31 to the tested antibiotics and other non-tested antibiotics.

To further explore the resistance mechanism of J31, we used PlasmidFinder to detect the potential plasmids among the whole genome sequence. However, PlasmidFinder did not find any plasmid, and the result was also verified by conjugation experiments. We further investigated the other three assemblies of *C. indologenes* in NCBI genome database (https://www.ncbi.nlm.nih.gov/genome/) and no typing plasmid replicons could be found in all the contigs. For the genus *Chryseobacterium*, only two in thirty-eight draft or complete genomes consist of chromosomes and plasmids, which suggests *Chryseobacterium*, unlike the genus *Enterococcus*, might carry the resistant genes in the chromosome [16, 29].

We scanned each ARG flanking sequences in a range of 10-kb for IS sequences and junction associated proteins. Only one partial IS sequence, 107 bp ISR of ISBbi1 in IS1595 family, was found locating at 8958 bp upstream of one subclass B1 MBL (Additional file 3: Table S3). We found two integrases, a 304-aa-long site-specific tyrosine recombinase XerD at 2908 bp downstream of the penicillin-binding protein gene and a 422-aa-long hypothetical integrase at 4146 bp downstream of the putative ABC transporter gene, and two crossover junction endodeoxyribonucleases, a 184-aa-long RuvC protein at 5578 bp downstream of one β-lactamase gene and a 138-aa-long RuvA protein at 1748 bp downstream of another β-lactamase gene (Additional file 3: Table S3). Maybe there are some unknown proteins flanking the predicted ARGs which might function as the insertion elements or transposases for transferring antibiotic resistance.

### A new IND-type MBL variant IND-16

We identified a novel IND-type MBL variant from in silico ARGs prediction, and designated it as IND-16. We confirmed this allele by PCR sequencing and searched the sequence against the GenBank database. The BLASTN search indicated the sequence was highly similar to *bla*_IND_ genes of *Chryseobacterium* species. Multiple sequence alignments demonstrated that IND-16 protein shared 99 % identity with IND-8 and IND-10 and was conserved with other IND type MBLs (Additional file 4: Figure S1). According to the distinctive resistance of the strain, IND-16 was likely to contribute to the resistance of J31 to carbapenems.

### Pathogenesis analysis

*Chryseobacterium indologenes* is an emerging pathogen associated with indwelling devices and immunosuppression, the pathogenesis should be investigated. We performed a BLASTP search against VRDB database and found three virulence factors which contained Clp protease ClpC, molecular chaperone GroEL and ATP-dependent chaperone ClpB (Additional file 5: Table S4). In addition, we detected four conjugative transposon clusters, Tra gene clusters, in the draft genomes which inferred the strain J31 was with potential pathogenesis (Additional file 6: Table S5).

### Comparative analysis with other *C. indologenes* strains

**Fig. 3:**
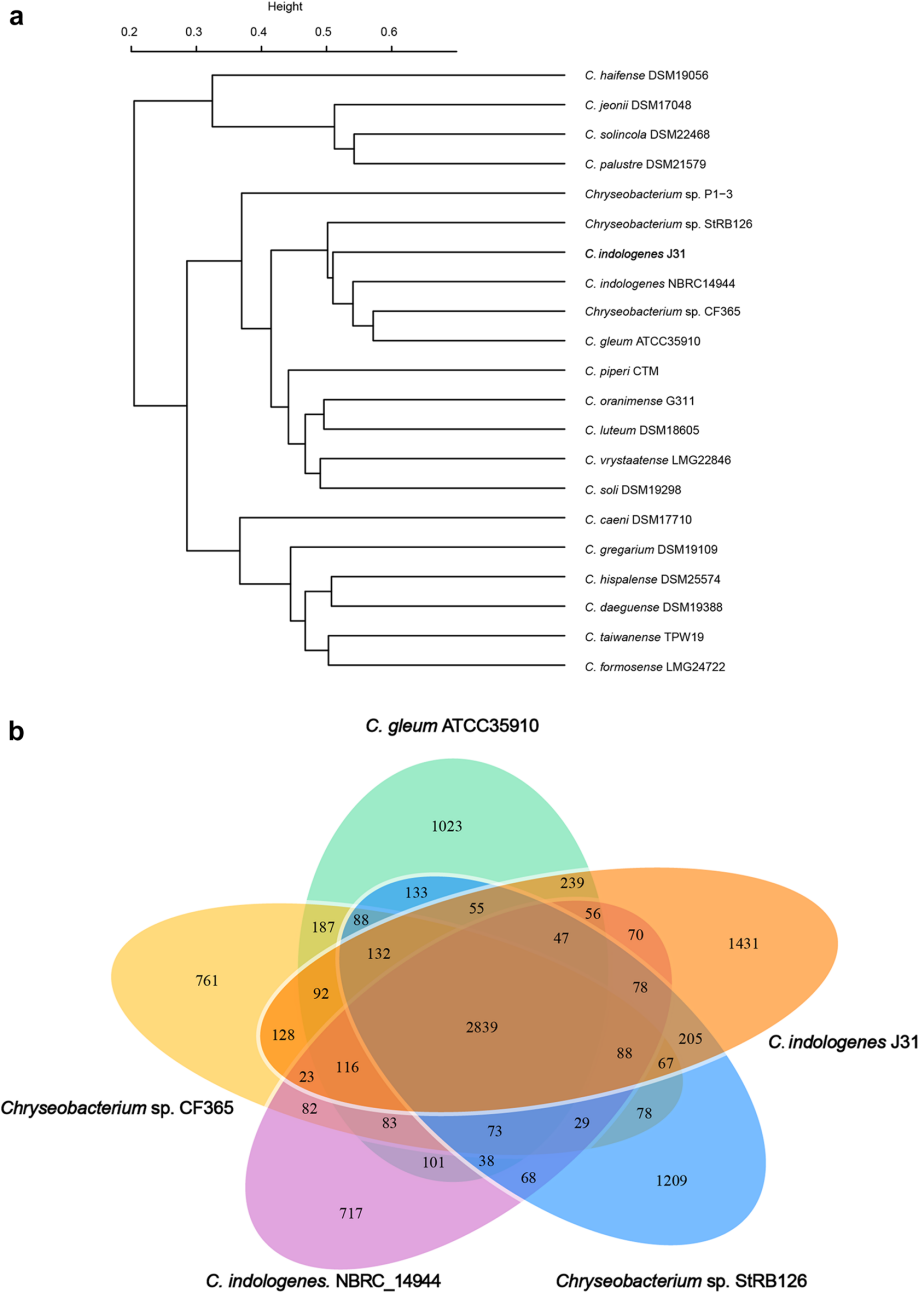
Genome phylogenetic tree and Venn diagrams of five *Chryseobacterium* genomes. **a** The whole genome phylogenetic analysis demonstrating the relationship between *C*. *indologene* J31 sequenced in this study and other 20 genome sequences from public databases (see Additional file 7: Table S6 for accessions). The tree was constructed based on core genome alignments. **b** *Venn diagrams* showing the orthologous groups in the five *Chryseobacterium* genomes. *Numbers* inside the *Venn diagrams* indicated the number of genes found to be shared among the indicated genomes

According to the comparison of whole genome, strain J31 presented a high conserved structure to other five *Chryseobacterium* species (Fig. 3). In the purpose of defining the evolution position of J31, we constructed the whole-genome phylogenetic tree by an all-against-all BLASTP comparison of the complete gene sets of J31 with twenty closely related *Chryseobacterium* species (Fig. 3a). Phylogenetic analysis revealed J31 was closely related to *Chryseobacterium sp.* StRB126 and *C. indologenes* NBRC14944. Among the closely four related neighbors of stain J31, we performed a comparison of functional genes. Venn diagram indicated the presence of 2839 core conserved genes present in the pan-genome of the genus *Chryseobacterium* (Fig. 3b). A great number of 1431 strain-specific genes were identified in strain J31. These findings imply that *Chryseobacterium* species showed significant differences, although they shared high similarity in whole genomic level, which was consistent with the multiple novel resistance determinants observed in J31.

### Future directions

This study represents the first genomic features of a multidrug resistant *C. indologenes* isolate and demonstrates the comparative genomic analysis of strain J31 to other *Chryseobacterium* species. Notably, it reveals the presence of numerous resistance determinants that helped this strain resistant to many antimicrobial agents, which makes it potential candidates for nosocomial infections. Furthermore, our analysis advances the understanding of resistance in genus *Chryseobacterium*.

## Additional files

**Additional files 1: Table S1.** Antimicrobial susceptibility profiles of *C. indologenes* J31.

**Additional files 2: Table S2.** Numerous antibiotic resistance genes predicted in the *C. indologenes* J31 genome.

**Additional files 3: Table S3.** Insertion sequence, integrase gene and transposase genes analysis.

**Additional files 4: Figure S1.** Multi-alignments of the protein sequence of IND-16 from *C. indologenes* J31 with other IND type MBLs. The IND variants used in the alignments are as follows: IND-1 (AF099139), IND-2 (AF219129), IND-3 (AF219133), IND-4 (AAG29765), IND-5 (AY504627), IND-6 (AM087455), IND-7 (BAJ05825), IND-8 (ACZ65152), IND-10 (ADA13241) and IND-16 (KT235893). Multiple alignments were performed using Clustal Omega and visualized by BoxShade.

**Additional files 5: Table S4.** Potential Virulence factors identified in the *Chryseobacterium indologenes* J31 genome.

**Additional files 6: Table S5.** Four conjugative transposon gene clusters in the *Chryseobacterium indologenes* J31 genome.

**Additional files 7: Table S6.** List of genome accessions used in whole-genome phylogenetic analysis.
