# Supplementary material for: Whole genome sequencing uncovers a novel IND-16 metallo-β-lactamase from an extensively drug-resistant Chryseobacterium indologenes strain J31
Source: Gut Pathog. 2016 Oct 21;8:47. doi: 10.1186/s13099-016-0130-4 (PMC5073886; doi:10.1186/s13099-016-0130-4)
Supplement: Supplementary file 1 — Additional files 1: Table S1. Antimicrobial susceptibility profiles of C. indologenes J31. [file 13099_2016_130_MOESM1_ESM.docx]

**Table S1.** Antimicrobial susceptibility profiles of *C. indologenes* J31.

| **Antimicrobial agent** | **MIC (μg/ml) ^a^** | **Susceptibility** |
| --- | --- | --- |
| Ampicillin | ≥32 | R |
| Amikacin | ≥64 | R |
| Ciprofloxacin | ≥4 | R |
| Levofloxacin | ≥8 | R |
| Cefoperazone | ≥64 | R |
| Nitrofurantoin | 128 | R |
| Imipenem | ≥16 | R |
| Trimethoprim-sulphamethoxazole | ≤2 | S |
| Tobramycin | ≥16 | R |
| Piperacillin-tazobactam | ≥128 | R |
| Ampicillin-sulbactam | ≥32 | R |
| Aztreonam | ≥64 | R |
| Cefotetan | ≥64 | R |
| Cefazolin | ≥64 | R |
| Gentamicin | ≥16 | R |
| Ceftazidime | ≥64 | R |
| Cefepime | ≥64 | R |
| Colistin | 32 | R |

^a^ MICs were determined using the Vitek 2 GN and AST cards, following the manufacturer’s instructions. Colistin MICs were determined by Etest according to the manufacturer's guidelines (Biomerieux, France). The MIC was read where inhibition of growth intersected the Etest strip. The susceptibility breakpoints of MICs followed those recommend by the Clinical and Laboratory Standards Institute for *Pseudomonas aeruginosa* and non-Enterobacteriaceae.
