# Supplementary material for: Whole genome sequencing uncovers a novel IND-16 metallo-β-lactamase from an extensively drug-resistant Chryseobacterium indologenes strain J31
Source: Gut Pathog. 2016 Oct 21;8:47. doi: 10.1186/s13099-016-0130-4 (PMC5073886; doi:10.1186/s13099-016-0130-4)
Supplement: Supplementary file 2 — Additional files 2: Table S2. Numerous antibiotic resistance genes predicted in the C. indologenes J31 genome. [file 13099_2016_130_MOESM2_ESM.doc]

**Table S2.** Numerous antibiotic resistance genes predicted in the *Chryseobacterium indologenes* J31 genome

| **Drug class** | **Gene product** | **Description** | **Best BLAST hit organism in GenBank** | **Amino acid identity (%)** |
| --- | --- | --- | --- | --- |
| β-lactams |  | β-lactamase class C | *[Chryseobacterium gleum](http://blast.ncbi.nlm.nih.gov/Blast.cgi#alnHdr_489067688)* (WP_002977677.1) | 373/495 (75%) |
|  |  | Metallo-β-lactamase | *Chryseobacterium* sp. CF365 ([WP_034699903.1](http://www.ncbi.nlm.nih.gov/protein/736694223?report=genbank&log$=prottop&blast_rank=1&RID=KER58ZNF01N)) | 195/252 (77%) |
|  | PenP | β-lactamase class A | *Chryseobacterium* sp. StRB126 ([WP_045497232.1](http://www.ncbi.nlm.nih.gov/protein/780164418?report=genbank&log$=prottop&blast_rank=1&RID=KEV3X7U2013)) | 234/295 (79%) |
|  |  | Metallo-β-lactamase | *Chryseobacterium* sp. StRB126 (WP_045497157.1) | 224/228 (98%) |
|  | IND-16 | Metallo-β-lactamase | *Chryseobacterium* *indologenes* NBRC 14944 ([ACZ65152.1](http://www.ncbi.nlm.nih.gov/protein/270266460?report=genbank&log$=prottop&blast_rank=1&RID=KEXCT94001N)) | 238/239 (99%) |
|  | CME-1 | β-lactamase class A | [*Chryseobacterium* sp. UNC8MFCol (WP_027373439.1](http://www.ncbi.nlm.nih.gov/protein/653124005?report=genbank&log$=prottop&blast_rank=1&RID=KEYAY5C5013)) | 265/292 (90%) |
|  |  | Metallo-β-lactamase superfamily | [*Chryseobacterium taiwanense* (WP_039365186.1](http://www.ncbi.nlm.nih.gov/protein/746318521?report=genbank&log$=prottop&blast_rank=1&RID=KEYVUXTX01N)) | 280/288 (97%) |
|  |  | β-lactamase superfamily | *[Chryseobacterium gleum](http://blast.ncbi.nlm.nih.gov/Blast.cgi#alnHdr_489067688)* ([WP_002983482.1](http://www.ncbi.nlm.nih.gov/protein/489073525?report=genbank&log$=prottop&blast_rank=1&RID=KF1U4ZYU013)) | 305/349 (87%) |
|  |  | Metallo-β-lactamase superfamily | *Chryseobacterium gleum* ATCC 35910 ([EFK36491.1](http://www.ncbi.nlm.nih.gov/protein/300505352?report=genbank&log$=prottop&blast_rank=1&RID=KF3PG4CX013)) | 200/212 (94%) |
|  | AmpC | β-lactamase class C | *Chryseobacterium gleum* ATCC 35910 ([EFK37196.1](http://www.ncbi.nlm.nih.gov/protein/300506060?report=genbank&log$=prottop&blast_rank=1&RID=KFAYGG2R015)) | 394/420 (94%) |
|  |  | β-lactamase class C | *Chryseobacterium gleum* ATCC 35910 ([WP_034696409.1](http://www.ncbi.nlm.nih.gov/protein/736690668?report=genbank&log$=prottop&blast_rank=1&RID=KFB0KHA6014)) | 266/324 (82%) |
|  |  | β-lactamase class C superfamily | *Chryseobacterium indologenes* NBRC 14944 ([GAE64579.1](http://www.ncbi.nlm.nih.gov/protein/573452410?report=genbank&log$=prottop&blast_rank=1&RID=KGRM36XG01R)) | 338/465 (73%) |
|  |  | β-lactamase class C superfamily | [*Chryseobacterium* sp. CF365 (WP_034696165.1](http://www.ncbi.nlm.nih.gov/protein/736690422?report=genbank&log$=prottop&blast_rank=1&RID=KH94P8ES01R)) | 379/535 (71%) |
|  |  | β-lactamase class C superfamily | *Flavobacterium johnsoniae* ([WP_012024175.1](http://www.ncbi.nlm.nih.gov/protein/500933234?report=genbank&log$=prottop&blast_rank=1&RID=KHX8WG22014)) | 178/328 (54%) |
|  |  | β-lactamase class C superfamily | *Chryseobacterium gleum* ([WP_041461369.1](http://www.ncbi.nlm.nih.gov/protein/752822794?report=genbank&log$=prottop&blast_rank=1&RID=KHY7T17U015)) | 325/369 (88%) |
|  |  | β-lactamase | *Chryseobacterium* sp. CF365 ([WP_034693372.1](http://www.ncbi.nlm.nih.gov/protein/736687619?report=genbank&log$=prottop&blast_rank=1&RID=KJ0GF0AT01R)) | 493/514 (96%) |
|  |  | β-lactamase | *Chryseobacterium* sp. CF365 ([WP_034698374.1](http://www.ncbi.nlm.nih.gov/protein/736692669?report=genbank&log$=prottop&blast_rank=1&RID=KK82E3W8014)) | 355/458 (78%) |
|  |  | Metallo-β-lactamase superfamily | *Chryseobacterium* sp. StRB126 ([BAP31609.1](http://www.ncbi.nlm.nih.gov/protein/674272216?report=genbank&log$=prottop&blast_rank=1&RID=KK9P0EY9014)) | 209/242 (86%) |
|  |  | Metallo-β-lactamase superfamily | *Chryseobacterium* sp. CF365 ([WP_034697277.1](http://www.ncbi.nlm.nih.gov/protein/736691545?report=genbank&log$=prottop&blast_rank=1&RID=KKARV6K2014)) | 287/344 (83%) |
|  | AmpC | β-lactamase class C superfamily | *Chryseobacterium* sp. P1-3 ([KFF75317.1](http://www.ncbi.nlm.nih.gov/protein/671763627?report=genbank&log$=prottop&blast_rank=1&RID=KM8SS5JH014)) | 311/356 (87%) |
|  |  | β-lactamase | *Chryseobacterium* sp. P1-3 ([KFF75566.1](http://www.ncbi.nlm.nih.gov/protein/671763876?report=genbank&log$=prottop&blast_rank=1&RID=KM92NDT6014)) | 399/497 (80%) |
|  |  | Metallo-β-lactamase superfamily | *Chryseobacterium* sp. StRB126 ([WP_045498117.1](http://www.ncbi.nlm.nih.gov/protein/780166538?report=genbank&log$=prottop&blast_rank=1&RID=KM990TSP014)) | 272/330 (82%) |
|  | AmpC | β-lactamase class C superfamily | *Chryseobacterium* sp. StRB126 ([WP_045499269.1](http://www.ncbi.nlm.nih.gov/protein/780169428?report=genbank&log$=prottop&blast_rank=1&RID=KMA6GBPM014)) | 307/352 (87%) |
|  |  | β-lactamase class C superfamily | *Myroides* sp. A21 ([WP_039328210.1](http://www.ncbi.nlm.nih.gov/protein/746280729?report=genbank&log$=prottop&blast_rank=1&RID=KMATA84R014)) | 117/183 (64%) |
|  | AmpC | β-lactamase class C superfamily | *Chryseobacterium* sp. StRB126 ([BAP32201.1](http://www.ncbi.nlm.nih.gov/protein/674272808?report=genbank&log$=prottop&blast_rank=1&RID=KMC3C3SD01R)) | 418/475 (88%) |
|  |  | β-lactamase class C superfamily | *Chryseobacterium* sp. StRB126 ([WP_045498583.1](http://www.ncbi.nlm.nih.gov/protein/780167748?report=genbank&log$=prottop&blast_rank=1&RID=KMCMY6VJ015)) | 205/468 (44%) |
|  |  | β-lactamase | *Chryseobacterium indologenes* NBRC 14944 ([WP_034737375.1](http://www.ncbi.nlm.nih.gov/protein/736733408?report=genbank&log$=prottop&blast_rank=1&RID=KMCXW4Z7015)) | 291/372 (78%) |
|  | AmpC | β-lactamase class C superfamily | *Chryseobacterium* sp. CF365 ([WP_034691664.1](http://www.ncbi.nlm.nih.gov/protein/736685906?report=genbank&log$=prottop&blast_rank=1&RID=KMD01FX0015)) | 311/354 (88%) |
|  |  | β-lactamase class C superfamily | *Elizabethkingia meningoseptica* ([WP_016198033.1](http://www.ncbi.nlm.nih.gov/protein/510824863?report=genbank&log$=prottop&blast_rank=1&RID=KMEYZWYU014)) | 240/306 (78%) |
|  |  | Metallo-β-lactamase superfamily | *Capnocytophaga* sp. oral taxon 324 ([WP_036676870.1](http://www.ncbi.nlm.nih.gov/protein/738785595?report=genbank&log$=prottop&blast_rank=1&RID=KMJYAHDG014)) | 125/295 (42%) |
|  | PBP2 | Penicillin-binding protein 2 | *Chryseobacterium indologenes* NBRC 14944 ([WP_034734578.1](http://www.ncbi.nlm.nih.gov/protein/736730440?report=genbank&log$=prottop&blast_rank=1&RID=KHXNMW39015)) | 626/647 (97%) |
| Rifampin | Arr-ms | Rifampin ADP-ribosyl transferase | *Chryseobacterium piperi* ([WP_034685286.1](http://www.ncbi.nlm.nih.gov/protein/736679024?report=genbank&log$=prottop&blast_rank=1&RID=KFCDP5RW014)) | 117/145 (81%) |
| Aminoglycosides | AadK | Aminoglycoside 6- adenylyltransferase | *Chryseobacterium* sp. CF365 ([WP_034693616.1](http://www.ncbi.nlm.nih.gov/protein/736687863?report=genbank&log$=prottop&blast_rank=1&RID=KF2GG5ZN01N)) | 233/287 (81%) |
| Phenicols | Cfr | Radical SAM superfamily | *Chryseobacterium* sp. StRB126 ([WP_045499089.1](http://www.ncbi.nlm.nih.gov/protein/780168999?report=genbank&log$=prottop&blast_rank=1&RID=KF7W5XSH01R)) | 337/344 (98%) |
|  | Flor | Drug resistance transporter | *Chryseobacterium gleum* ATCC 35910 ([EFK34307.1](http://www.ncbi.nlm.nih.gov/protein/300503167?report=genbank&log$=prottop&blast_rank=1&RID=KH7PKM8K015)) | 378/402 (94%) |
|  | Flor | Drug resistance transporter | *[Chryseobacterium](http://blast.ncbi.nlm.nih.gov/Blast.cgi#alnHdr_671762258)* sp. P1-3 ([KFF73954.1](http://www.ncbi.nlm.nih.gov/protein/671762258?report=genbank&log$=prottop&blast_rank=1&RID=KHC2BJK2014)) | 360/395 (91%) |
| Trimethoprim | DfrA | Dihydrofolate reductase | *Chryseobacterium* sp. CF365 ([WP_034693339.1](http://www.ncbi.nlm.nih.gov/protein/736687586?report=genbank&log$=prottop&blast_rank=1&RID=KFD6SMJN015)) | 153/165 (93%) |
| Su1phonamide | SulII | Dihydropteroate synthase | *Chryseobacterium* sp. StRB126 ([WP_045494645.1](http://www.ncbi.nlm.nih.gov/protein/780157556?report=genbank&log$=prottop&blast_rank=1&RID=M1RVW7NX014)) | 239/279 (86%) |
| Macrolide | MacA | Macrolide transporter subunit | *Chryseobacterium gleum* ([WP_002981305.1](http://www.ncbi.nlm.nih.gov/protein/489071338?report=genbank&log$=prottop&blast_rank=1&RID=KPMSAH2S01R)) | 371/408 (91%) |
|  | MacB | Macrolide transporter ATP-binding /permease protein | *Chryseobacterium* sp. CF365 ([WP_034700218.1](http://www.ncbi.nlm.nih.gov/protein/736694550?report=genbank&log$=prottop&blast_rank=1&RID=KPN3PN59015)) | 388/409(95%) |
| Multidrug efflux  pumps | EmrB | MFS superfamily efflux transporter | *Chryseobacterium* sp. CF365 ([WP_034699938.1](http://www.ncbi.nlm.nih.gov/protein/736694258?report=genbank&log$=prottop&blast_rank=1&RID=KEN0AY1S016)) | 505/513 (98%) |
|  | EmrA | MFS superfamily efflux transporter | [*Chryseobacterium* sp. CF365 (WP_034699936.1](http://www.ncbi.nlm.nih.gov/protein/736694256?report=genbank&log$=prottop&blast_rank=1&RID=KENBZRGH016)) | 362/367 (99%) |
|  | EmrD | MFS superfamily efflux transporter | *Chryseobacterium* sp. CF365 ([WP_034695347.1](http://www.ncbi.nlm.nih.gov/protein/736689600?report=genbank&log$=prottop&blast_rank=1&RID=KETYG2NY01N)) | 300/380 (79%) |
|  | TolC | Outer membrane efflux protein | *Chryseobacterium* sp. CF365 ([WP_034699934.1](http://www.ncbi.nlm.nih.gov/protein/736694254?report=genbank&log$=prottop&blast_rank=1&RID=KEP712FP01N)) | 418/431 (97%) |
|  | AcrA | RND family efflux transporter | *[Chryseobacterium gleum](http://blast.ncbi.nlm.nih.gov/Blast.cgi#alnHdr_489067688)* ([EFK36895.1](http://www.ncbi.nlm.nih.gov/protein/300505758?report=genbank&log$=prottop&blast_rank=1&RID=KERS0STH01N)) | 360/361 (99%) |
|  | MdlB | ABC-type multidrug transport system | *Chryseobacterium* sp. CF365 ([WP_034695352.1](http://www.ncbi.nlm.nih.gov/protein/736689605?report=genbank&log$=prottop&blast_rank=1&RID=KETZ9P1201N)) | 579/600 (97%) |
|  | AcrA | RND family efflux transporter | *Spirosoma panaciterrae* ([WP_020596138.1](http://www.ncbi.nlm.nih.gov/protein/522084929?report=genbank&log$=prottop&blast_rank=1&RID=KEZ5RMYE013)) | 154/256 (60%) |
|  |  | RND family efflux transporter | *Chryseobacterium indologenes* NBRC 14944 ([WP_034735417.1](http://www.ncbi.nlm.nih.gov/protein/736731332?report=genbank&log$=prottop&blast_rank=1&RID=KF3BWWMP016)) | 593/627 (95%) |
|  | AcrA | RND family efflux transporte | *Chryseobacterium* sp. P1-3 ([KFF75231.1](http://www.ncbi.nlm.nih.gov/protein/671763541?report=genbank&log$=prottop&blast_rank=1&RID=KF48JWJP01N)) | 359/386 (93%) |
|  | AcrA | RND family efflux transporter | *Chryseobacterium gleum* ATCC 35910 ([EFK37935.1](http://www.ncbi.nlm.nih.gov/protein/300506800?report=genbank&log$=prottop&blast_rank=1&RID=KF65695S01R)) | 347/371 (94%) |
|  | AcrA | RND family efflux transporter | *Chryseobacterium* sp. CF365 ([WP_034696931.1](http://www.ncbi.nlm.nih.gov/protein/736691194?report=genbank&log$=prottop&blast_rank=1&RID=KF7G4819015)) | 352/381 (92%) |
|  | YhcA | Multidrug resistance transporter | *Chryseobacterium* sp. CF365 ([WP_034696928.1](http://www.ncbi.nlm.nih.gov/protein/736691191?report=genbank&log$=prottop&blast_rank=1&RID=KF7MNU3A014)) | 422/461 (92%) |
|  |  | [M](http://blast.ncbi.nlm.nih.gov/Blast.cgi#alnHdr_736690663)ultidrug ABC transporter ATPase | *Chryseobacterium* sp. CF365 ([WP_034696404.1](http://www.ncbi.nlm.nih.gov/protein/736690663?report=genbank&log$=prottop&blast_rank=1&RID=KF8AVZZZ014)) | 222/231 (96%) |
|  | AcrB | RND family efflux transporter | *Chryseobacterium* sp. CF365 ([WP_034699255.1](http://www.ncbi.nlm.nih.gov/protein/736693561?report=genbank&log$=prottop&blast_rank=1&RID=KGN9SKUW015)) | 993/1035 (96%) |
|  | AcrA | RND family efflux transporter | *Chryseobacterium indologenes* NBRC 14944 ([WP_034735418.1](http://www.ncbi.nlm.nih.gov/protein/736731333?report=genbank&log$=prottop&blast_rank=1&RID=KGPZ1KAD01R)) | 321/367 (87%) |
|  | MdlB | ABC-type multidrug transport system | *Chryseobacterium* sp. P1-3 ([KFF74169.1](http://www.ncbi.nlm.nih.gov/protein/671762474?report=genbank&log$=prottop&blast_rank=1&RID=KGST6XTM014)) | 584/612 (95%) |
|  | MdlB | ABC-type multidrug transport system | *Chryseobacterium* sp. StRB126 ([BAP33434.1](http://www.ncbi.nlm.nih.gov/protein/674274041?report=genbank&log$=prottop&blast_rank=1&RID=KH8TNVPS015)) | 516/541 (95%) |
|  | CcmA | ABC-type multidrug transport system | *Chryseobacterium* sp. UNC8MFCol ([WP_027375052.1](http://www.ncbi.nlm.nih.gov/protein/653125638?report=genbank&log$=prottop&blast_rank=1&RID=KHA7909M01R)) | 261/290 (90%) |
|  | AcrA | RND family efflux transporter | *Chryseobacterium gleum* ([WP_002982259.1](http://www.ncbi.nlm.nih.gov/protein/489072301?report=genbank&log$=prottop&blast_rank=1&RID=KHC1D95M015)) | 336/373 (90%) |
|  | CcmA | ABC-type multidrug transport system | *Flavobacterium sasangense* ([WP_026726564.1](http://www.ncbi.nlm.nih.gov/protein/652329312?report=genbank&log$=prottop&blast_rank=1&RID=KHCN0X9W01R)) | 213/379 (56%) |
|  | AcrB | RND family efflux transporter | *Chryseobacterium* sp. StRB126 ([WP_045497730.1](http://www.ncbi.nlm.nih.gov/protein/780165556?report=genbank&log$=prottop&blast_rank=1&RID=KHEPE0SY01R)) | 999/1029 (97%) |
|  | YhcA | Multidrug resistance transporter | *Chryseobacterium* sp. CF365 ([WP_034697011.1](http://www.ncbi.nlm.nih.gov/protein/736691275?report=genbank&log$=prottop&blast_rank=1&RID=KHG16XHK01R)) | 449/467 (96%) |
|  | AcrA | RND family efflux transporter | *Chryseobacterium* sp. CF365 ([WP_034693169.1](http://www.ncbi.nlm.nih.gov/protein/736687415?report=genbank&log$=prottop&blast_rank=1&RID=KJ1130NB014)) | 349/361 (97%) |
|  | AcrB | RND family efflux transporter | *Chryseobacterium gleum* ([WP_002976404.1](http://www.ncbi.nlm.nih.gov/protein/489066406?report=genbank&log$=prottop&blast_rank=1&RID=KJ11D1M4014)) | 977/996 (98%) |
|  | AcrA | RND family efflux transporter | *Chryseobacterium* sp. StRB126 ([WP_045496391.1](http://www.ncbi.nlm.nih.gov/protein/780162272?report=genbank&log$=prottop&blast_rank=1&RID=KJ29T1Y9014)) | 378/395 (96%) |
|  | AcrB | RND family efflux transporter | *Chryseobacterium* sp. CF365 ([WP_034694383.1](http://www.ncbi.nlm.nih.gov/protein/736688632?report=genbank&log$=prottop&blast_rank=1&RID=KJ2CM3MV015)) | 1028/1048 (98%) |
|  | AcrA | RND family efflux transporter | *Chryseobacterium* sp. CF365 ([WP_034692131.1](http://www.ncbi.nlm.nih.gov/protein/736686375?report=genbank&log$=prottop&blast_rank=1&RID=KMCB22RY014)) | 327/346 (95%) |
|  | YhcA | Multidrug resistance transporter | *Chryseobacterium indologenes* NBRC 14944 ([WP_034736494.1](http://www.ncbi.nlm.nih.gov/protein/736732476?report=genbank&log$=prottop&blast_rank=1&RID=KMG8YJ30014)) | 348/457 (76%) |
|  | YhcA | Multidrug resistance transporter | *Chryseobacterium* sp. StRB126 ([WP_045500450.1](http://www.ncbi.nlm.nih.gov/protein/780172317?report=genbank&log$=prottop&blast_rank=1&RID=KMJPKGZT015)) | 332/446 (74%) |
|  | MatE | Multidrug transporter | *Chryseobacterium gleum* ATCC 35910 ([WP_002979924.1](http://www.ncbi.nlm.nih.gov/protein/489069950?report=genbank&log$=prottop&blast_rank=1&RID=KPBKX9AP014)) | 441/458 (96%) |
|  | OprM | RND fflux transporter | *Chryseobacterium* sp. StRB126 ([WP_045494309.1](http://www.ncbi.nlm.nih.gov/protein/780156776?report=genbank&log$=prottop&blast_rank=1&RID=KPCSU2WF015)) | 447/471 (95%) |
|  | NorM | Multidrug efflux pump | Chryseobacterium sp. P1-3 ([KFF74650.1](http://www.ncbi.nlm.nih.gov/protein/671762957?report=genbank&log$=prottop&blast_rank=1&RID=KPCE4ZRN01R)) | 400/446 (90%) |
|  | AcrB | RND family efflux transporter | *Chryseobacterium* sp. CF365 ([WP_034692132.1](http://www.ncbi.nlm.nih.gov/protein/736686376?report=genbank&log$=prottop&blast_rank=1&RID=KF0WSRTY013)) | 1023/1040 (98%) |
|  | OprM | RND efflux transporter | *Chryseobacterium* sp. StRB126 ([WP_045500766.1](http://www.ncbi.nlm.nih.gov/protein/780173138?report=genbank&log$=prottop&blast_rank=1&RID=KPCX4R0R01R)) | 450/470 (96%) |
|  | AcrB | RND family efflux transporter | *Chryseobacterium gleum* ([WP_002976721.1](http://www.ncbi.nlm.nih.gov/protein/489066725?report=genbank&log$=prottop&blast_rank=1&RID=KGNPZ8KK014)) | 1043/1060 (98%) |
|  | TolC | RND efflux transporter | *Chryseobacterium* sp. CF365 ([WP_034696961.1](http://www.ncbi.nlm.nih.gov/protein/736691224?report=genbank&log$=prottop&blast_rank=1&RID=KPDBMHG801R)) | 424/470 (90%) |
|  | AcrB | RND family efflux transporter | *Chryseobacterium* sp. CF365 ([WP_034696932.1](http://www.ncbi.nlm.nih.gov/protein/736691195?report=genbank&log$=prottop&blast_rank=1&RID=KPJX9HJF015)) | 1013/1046 (97%) |
|  | TolC | RND efflux transporter | *Chryseobacterium gleum* ([WP_002984690.1](http://www.ncbi.nlm.nih.gov/protein/489074738?report=genbank&log$=prottop&blast_rank=1&RID=KPDU0Y8D014)) | 442/457 (97%) |
|  | AcrB | RND family efflux transporter | *Chryseobacterium gleum* ([WP_002984692.1](http://www.ncbi.nlm.nih.gov/protein/489074740?report=genbank&log$=prottop&blast_rank=1&RID=KPDV0DJ6014)) | 1048/1063 (99%) |
|  | OprM | RND efflux transporter | *Chryseobacterium* sp. CF365 ([WP_034691940.1](http://www.ncbi.nlm.nih.gov/protein/736686183?report=genbank&log$=prottop&blast_rank=1&RID=KPGKH4TM01R)) | 443/471(94%) |
|  | AcrB | RND family efflux transporter | *Chryseobacterium* sp. StRB126 ([WP_045491940.1](http://www.ncbi.nlm.nih.gov/protein/780149165?report=genbank&log$=prottop&blast_rank=1&RID=KHBTKXJ4014)) | 1025/1052 (97%) |
|  | MFS | Major Facilitator Superfamily | *Chryseobacterium* sp. StRB126 ([BAP30414.1](http://www.ncbi.nlm.nih.gov/protein/674271021?report=genbank&log$=prottop&blast_rank=1&RID=M11S0GNM015)) | 368/400 (92%) |
|  | MFS | Major Facilitator Superfamily | *Chryseobacterium* sp. StRB126 ([WP_045496028.1](http://www.ncbi.nlm.nih.gov/protein/780161235?report=genbank&log$=prottop&blast_rank=1&RID=M12A32WP01R)) | 344/399 (86%) |
|  | MFS | Major Facilitator Superfamily | *Chryseobacterium* sp. CF365 ([WP_034694946.1](http://www.ncbi.nlm.nih.gov/protein/736689197?report=genbank&log$=prottop&blast_rank=1&RID=M12V58V2015)) | 388/409 (95%) |
|  | MFS | Major Facilitator Superfamily | *Chryseobacterium* sp. UNC8MFCol ([WP_027375175.1](http://www.ncbi.nlm.nih.gov/protein/653125762?report=genbank&log$=prottop&blast_rank=1&RID=M131C2RP015)) | 321/386 (83%) |
|  | MFS | Major Facilitator Superfamily | *Chryseobacterium* sp. UNC8MFCol ([WP_027375175.1](http://www.ncbi.nlm.nih.gov/protein/653125762?report=genbank&log$=prottop&blast_rank=1&RID=M15668VW014)) | 396/413 (96%) |
|  | MFS | Major Facilitator Superfamily | *Chryseobacterium* sp. CF365 ([WP_034696282.1](http://www.ncbi.nlm.nih.gov/protein/736690540?report=genbank&log$=prottop&blast_rank=1&RID=M14WJEWH014)) | 395/414 (95%) |
|  | EmrE | Small Multidrug Resistance protein | *Chryseobacterium* sp. CF365 ([WP_034697067.1](http://www.ncbi.nlm.nih.gov/protein/736691332?report=genbank&log$=prottop&blast_rank=1&RID=M1TXPJT601R)) | 109/111 (98%) |
