# Supplementary figures and images for: Whole genome sequencing uncovers a novel IND-16 metallo-β-lactamase from an extensively drug-resistant Chryseobacterium indologenes strain J31
Source: Gut Pathog. 2016 Oct 21;8:47. doi: 10.1186/s13099-016-0130-4 (PMC5073886; doi:10.1186/s13099-016-0130-4)

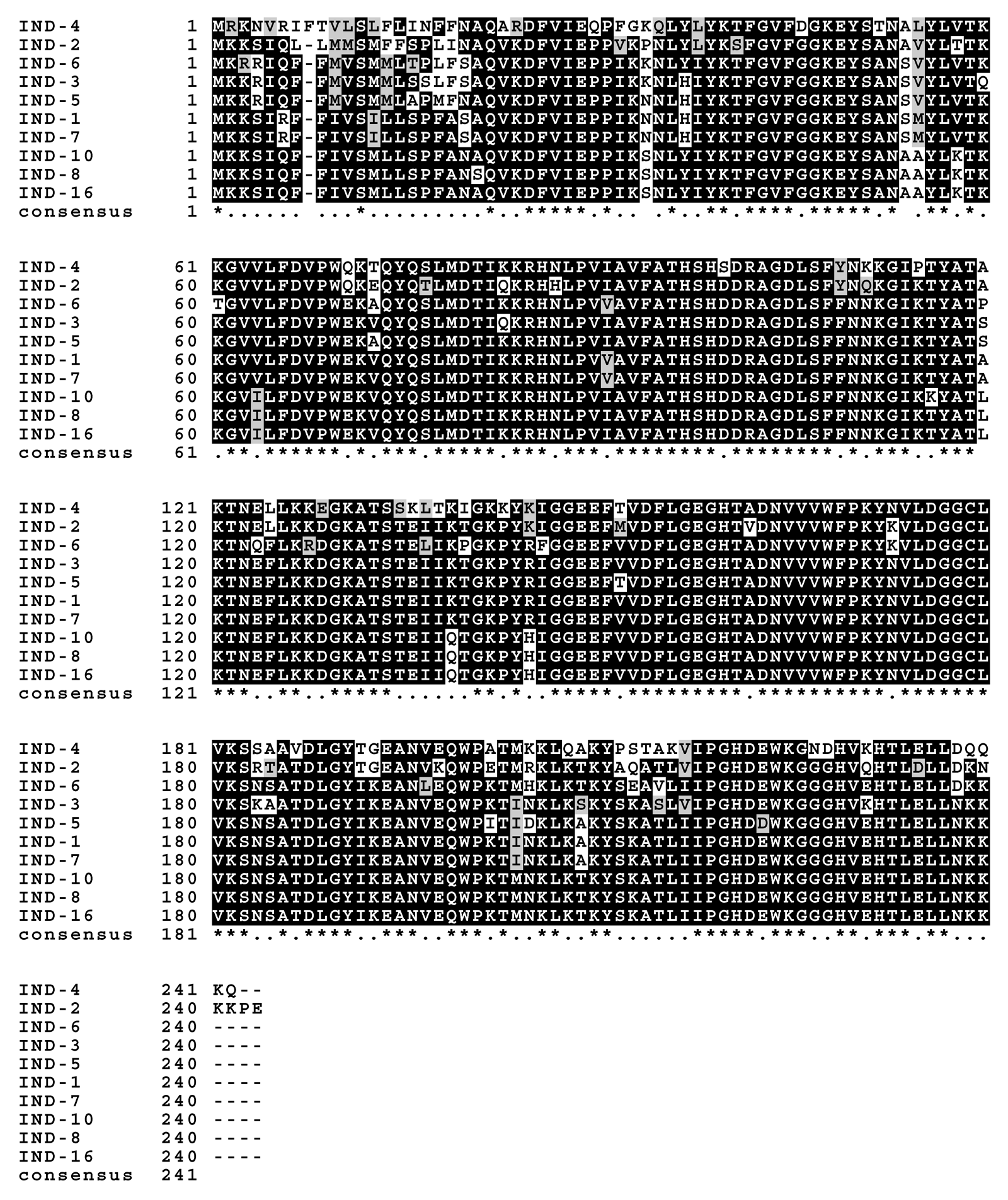

Supplement: Supplementary file 4 — Additional files 4: Figure S1. Multi-alignments of the protein sequence of IND-16 from C. indologenes J31 with other IND type MBLs. The IND variants used in the alignments are as follows: IND-1 (AF099139), IND-2 (AF219129), IND-3 (AF219133), IND-4 (AAG29765), IND-5 (AY504627), IND-6 (AM087455), IND-7 (BAJ05825), IND-8 (ACZ65152), IND-10 (ADA13241) and IND-16 (KT235893). Multiple alignments were performed using Clustal Omega and visualized by BoxShade. [file 13099_2016_130_MOESM4_ESM.tif]
