# Supplementary material for: Whole genome sequencing uncovers a novel IND-16 metallo-β-lactamase from an extensively drug-resistant Chryseobacterium indologenes strain J31
Source: Gut Pathog. 2016 Oct 21;8:47. doi: 10.1186/s13099-016-0130-4 (PMC5073886; doi:10.1186/s13099-016-0130-4)
Supplement: Supplementary file 5 — Additional files 5: Table S4. Potential Virulence factors identified in the Chryseobacterium indologenes J31 genome. [file 13099_2016_130_MOESM5_ESM.docx]

**Table S4.** Potential Virulence factors identified in the *Chryseobacterium indologenes* J31 genome.

| **Description** | **Best BLAST hit organism in GenBank** | **Amino acid identity (%)** |
| --- | --- | --- |
| [Clp protease, ClpC](https://blast.ncbi.nlm.nih.gov/Blast.cgi#alnHdr_736688475) | [[Chryseobacterium sp. CF365]](https://blast.ncbi.nlm.nih.gov/Blast.cgi#alnHdr_736690398)  [WP_034694227.1](https://www.ncbi.nlm.nih.gov/protein/736688475?report=genbank&log$=prottop&blast_rank=2&RID=WT56HB74014) | 99% |
| Molecular chaperone, GroEL | [[Chryseobacterium sp. CF365]](https://blast.ncbi.nlm.nih.gov/Blast.cgi#alnHdr_736690398)  [WP_034696141.1](https://www.ncbi.nlm.nih.gov/protein/736690398?report=genbank&log$=prottop&blast_rank=2&RID=WT56HB74014) | 99% |
| [ATP-dependent chaperone, ClpB](https://blast.ncbi.nlm.nih.gov/Blast.cgi#alnHdr_823662657) | [[Chryseobacterium sp. CF365]](https://blast.ncbi.nlm.nih.gov/Blast.cgi#alnHdr_736690398)  [WP_034695785.1](https://www.ncbi.nlm.nih.gov/protein/736690040?report=genbank&log$=prottop&blast_rank=2&RID=WT56HB74014) | 98% |
