# Supplementary material for: Whole genome sequencing uncovers a novel IND-16 metallo-β-lactamase from an extensively drug-resistant Chryseobacterium indologenes strain J31
Source: Gut Pathog. 2016 Oct 21;8:47. doi: 10.1186/s13099-016-0130-4 (PMC5073886; doi:10.1186/s13099-016-0130-4)
Supplement: Supplementary file 6 — Additional files 6: Table S5. Four conjugative transposon gene clusters in the Chryseobacterium indologenes J31 genome. [file 13099_2016_130_MOESM6_ESM.docx]

**Table S4.** Four conjugative transposon gene clusters in the *Chryseobacterium indologenes* J31 genome

|  | **Location** | **Conjugative transposon genes (position)** | **Predicted genes (position)** |
| --- | --- | --- | --- |
| Cluster 1 | Contig_10 | TraQ (203620_203171),  TraO (204192_203632),  TraN (205112_204213),  TraM (206477_205137),  TraK (207831_207208),  TraJ (208090_207851) |  |
| Cluster 2 | Contig_19 | TraE (373_50),  TraA (1739_1089) | BF0132 (3169_1802) |
| Cluster 3 | Contig_62 | TraN (25881_25015),  TraM (27088_25886),  TraK (28092_27478),  TraJ (29226_28126),  TraG (35278_32204) |  |
| Cluster 4 | Contig_71 | TraI (1107_475),  TraG (3640_1136),  TraF (3966_3637),  TraE (4283_3978),  TraD (9177_8542),  TraB (9638_9189),  TraA (10410_9643) | BF0131 (11175_11612), BF0132 (11615_12898) |
