# Supplementary material for: Whole genome sequencing uncovers a novel IND-16 metallo-β-lactamase from an extensively drug-resistant Chryseobacterium indologenes strain J31
Source: Gut Pathog. 2016 Oct 21;8:47. doi: 10.1186/s13099-016-0130-4 (PMC5073886; doi:10.1186/s13099-016-0130-4)
Supplement: Supplementary file 7 — Additional files 7: Table S6. List of genome accessions used in whole-genome phylogenetic analysis. [file 13099_2016_130_MOESM7_ESM.docx]

**Table S3.** List of genome accessions used in whole-genome phylogenetic analysis

| **Accessions** | **Strains** |
| --- | --- |
| AUFK01000000 | *Chryseobacterium caeni* DSM17710.1 |
| AUMT01000000 | *Chryseobacterium daeguense* DSM19388.1 |
| JPRP01000000 | *Chryseobacterium formosense* LMG24722 |
| ACKQ02000000 | *Chryseobacterium gleum* ATCC35910 |
| AUMU01000000 | *Chryseobacterium gregarium* DSM19109.1 |
| JASZ01000000 | *Chryseobacterium haifense* DSM19056 |
| JARQ01000000 | *Chryseobacterium hispalense* DSM25574 |
| BAVL01000000 | *Chryseobacterium indologenes* NBRC14944 |
| JSYL01000000 | *Chryseobacterium jeonii* DSM17048 |
| JPRO01000000 | *Chryseobacterium luteum* DSM18605 |
| CDHM01000000 | *Chryseobacterium oranimense* G311 |
| AULL01000000 | *Chryseobacterium palustre* DSM21579.1 |
| JPRJ01000000 | *Chryseobacterium piperi* CTM |
| JPRH01000000 | *Chryseobacterium soli* DSM19298 |
| JSYK01000000 | *Chryseobacterium solincola* DSM22468 |
| JQJM01000000 | *Chryseobacterium sp.* CF365 |
| JPEQ01000000 | *Chryseobacterium sp.* P1-3 |
| NZ_AP014624.1 | *Chryseobacterium sp.* StRB126 |
| JWTA01000000 | *Chryseobacterium taiwanense* TPW19 |
| JPRI01000000 | *Chryseobacterium vrystaatense* LMG22846 |
